# The swimming trace *Undichna* from the latest Devonian Hangenberg Sandstone equivalent of Morocco

**DOI:** 10.1186/s13358-021-00237-9

**Published:** 2021-09-13

**Authors:** Christian Klug, Abdelouahed Lagnaoui, Melina Jobbins, Wahiba Bel Haouz, Amine Najih

**Affiliations:** 1 Paläontologisches Institut Und Museum, Universität Zürich, Karl-Schmid-Strasse 4, 8006 Zurich, Switzerland; 2 Interdisciplinary Research Laboratory in Sciences, Education and Training, Higher School of Education and Training Berrechid (ESEFB), Hassan First University, Route de Casablanca Km 3.5, BP 539, 26100 Berrechid, Grand-Casablanca Morocco; 3 Working-Team On Geology of Mineral and Energy Resources, Research Laboratory Physico-Chemistry of Processes and Materials, Faculty of Sciences and Techniques, Hassan First University of Settat, Route de Casablanca Km 3.5, BP 539, 26000 Settat, Grand-Casablanca Morocco; 4 Laboratory of Stratigraphy of Oil-and-Gas Bearing Reservoirs, Department of Paleontology and Stratigraphy, Institute of Geology and Petroleum Technologies, Kazan (Volga Region) Federal University, Kremlyovskaya Str. 18, Kazan, 420008 Russian Federation; 5 Institut National Supérieur du Professorat Et de L’Éducation de L’Académie de Poitiers, Université de Poitiers, 5 Rue Shirin Ebadi, 86073 Poitiers, France

**Keywords:** Vertebrata, Romer’s gap, Ichnofossils, Devonian, Palaeoecology

## Abstract

Trace fossils occur in several strata of the Devonian and Carboniferous of the eastern Anti-Atlas, but they are still poorly documented. Here, we describe a fossil swimming trace from strata overlying the Hangenberg Black Shale (correlation largely based on lithostratigraphy; *Postclymenia* ammonoid genozone, ca. 370 Ma old). We discuss the systematic position of the tracemaker and its body size. This ichnofossil is important for three main reasons: (1) it is considered here to be the first record of *Undichna* from the Devonian of Gondwana, as far as we know; (2) it is the oldest record of vertebrate trace fossils from Africa; (3) it provides a unique window into the behaviour of Late Devonian fishes for which body-fossils cannot provide direct evidence. Further, we put this discovery into the macroecological context of the palaeoenvironment following the Late Devonian Hangenberg biodiversity crisis.

## Introduction

Swimming traces of fishes such as *Undichna* are astonishingly rare, especially when taking into account that many of these animals live close to the sediment surface. Even more astonishingly, the oldest records of the ichnogenus *Undichna* date back to the Late Silurian (Knaust & Minter, 2018: *U. unisulca*). However, this ichnotaxon, *U. unisulca*, is not undulating like all other ichnospecies of *Undichna*; these ichnofossils range among the oldest published records of vertebrate traces apart from coprolites. Currently, the oldest sinusoidal-shaped *Undichna* and similar fish swimming traces are recorded from the Early Devonian (Morrisey et al., 2004; Trewin & Davidson, 1996; Wisshak et al., 2004), whereas the origin of vertebrates and fish-like chordates goes back much further (e.g., Brazeau, 2009; Brazeau & Friedman, 2015; Janvier, 1998; Zhu et al., 2013). While early chordates such as the Cambrian *Yunnanozoon*, *Haikouichthys* and *Pikaia* (Chen et al., 1995; Shu et al., 1996, 1999) appear unlikely to have produced swimming traces such as *Undichna*, many early gnathostomes with paired fins (e.g., Botella et al., 2007; Choo et al., 2014, 2017; Zhu et al., 2009) could well have performed swimming movements including body undulation with fins trailing on the sediment surface.

Even in the middle Palaeozoic or in younger strata, described occurrences of *Undichna* and similar ichnogenera such as the amphibian traces *Lunichnium* and *Serpentichnus* are rare. Both latter ichnotaxa display a combination of continuous and/ or discontinuous sinusoidal trails associated with scattered partial or complete footprints (Braddy et al., 2003; Minter & Braddy, 2006; Turek, 1989; Walter, 1983). Such ichnofossils are known from the Carboniferous of Argentina (Buatois & Mangano, 1994; Melchor & Cardonatto, 1998), Czech Republic (Turek, 1989, 1996), England (Higgs, 1988), Spain (Soler-Gijon & Moratalla, 2001), and USA (Martin, 2003; Martin & Rindsberg, 2004; Martin et al., 2010), the Permian of the Falkland Island (Trewin, 2000), South Africa (Anderson, 1970, 1976) and USA (Minter & Braddy, 2006), the Triassic of China (Lu & Chen, 1998; Lu et al., 2004), Germany (Simon et al., 2003), Italy (Todesco & Avanzini, 2008), and South Africa (Sciscio et al., 2020), the Jurassic of Germany (Schweigert, 2001) and USA (Gibert, 2001), as well as the Cretaceous of Spain (Gibert et al., 1999, 2000, 2001). Although this list is likely incomplete, we assume that not many references are missing here; this shows the scarcity of records, which might be partially to blame on the low number of active ichnologists. Consequently, every new record of *Undichna* is interesting and relevant.

The ichnogenus *Undichna* was formally introduced by Anderson (1976) for a Permian ichnofossil from South Africa. The ichnospecies included in *Undichna* consist of one or a combination of several sinusoidal furrows, which are commonly preserved as fillings, i.e. as hyporeliefs [for a definition see, e.g., Minter and Braddy (2006)]. More recently, Minter and Braddy (2006) published an ichnotaxonomic revision of the ichnogenus *Undichna*. They reduced the number of valid ichnospecies to nine: *U. simplicitas* Anderson, 1976, *U. bina* Anderson, 1976, *U. insolentia* Anderson, 1976, *U. britannica* Higgs, 1988, *U. consulca* Higgs, 1988, *U. unisulca* Gibert et al., 1999, *U. quina* Trewin, 2000, *U. trisulcata* Morrissey et al., 2004, *U. septemsulcata* Wisshak et al., 2004, and *U. unisulca* Knaust & Minter, 2018. Their revised ichnotaxonomy uses the number of furrows, how they undulate (in parallel, crossing), their continuity or discontinuity, paired or unpaired, and the depth of the furrow. As shown by Bainbridge (1958, 1963), Videler (1993) and Wisshak et al. (2004), amplitude, wavelength and relative course of the furrows allow to estimate the body size of the tracemaker.

Here, we describe a well-preserved *Undichna* from the latest Devonian of the southern Tafilalt region (Eastern Anti-Atlas, Morocco). In Morocco, sediments from all main global events of the Late Devonian have been documented. For example, deposits of the Kellwasser Events are excellently exposed (e.g., Wendt & Belka, 1991) and have yielded important data to improve our understanding of this mass extinction event (Buggisch, 1991; Buggisch & Joachimski, 2006; Hüneke, 2005). Similarly, the Famennian Annulata-Events (e.g., Hartenfels & Becker, 2016; Korn, 2004) as well as the Hangenberg Event are quite well studied in Morocco (Kaiser et al., 2006, 2011, 2013; Klug et al., 2016). Nevertheless, the outcrops are so vast and the strata locally so fossiliferous that they will yield plenty of materials for future studies for the next decades if not centuries.

The specimen presented here is interesting because the host layers overlie the supposed chronostratigraphic equivalent of the German Hangenberg Black Shale and are likely correlatable with the Hangenberg Sandstone. The Hangenberg Event (e.g., Algeo et al., 2001; Kaiser et al., 2006, 2011; Klug et al., 2016; Sandberg et al., 2002) particularly affected the vertebrate communities (Frey et al., 2018; Sallan & Galimberti, 2015). In many regions worldwide, a combination of a black shale (Hangenberg Black Shale) and a sandstone (Hangenberg Sandstone) is found. This has been interpreted (e.g., Kaiser et al., 2011) as a rapid change from a eustatic transgression (sedimentation of the Hangenberg Black Shale) to a regression (Hangenberg Sandstone), although, at least in Morocco, the Hangenberg Black Shale locally contains algae and other indicators of shallow water (Klug et al., 2016). According to the absence or extreme scarcity of vertebrate remains, the term Romer’s Gap was introduced for the interval between the Hangenberg Event and the late Visean because of the seeming lack (and actual scarcity) of tetrapod fossils (Coates & Clack, 1995; Romer, 1956; Smithson et al. 2012). However, vertebrate remains are also extremely scarce in the interval between the Hangenberg Black Shale [*Postclymenia* ammonoid genozone, middle *praesulcata* conodont zone, ca. 360 Ma; Becker et al. (2020); Kaiser et al. (2011)] and the end of the Carboniferous sedimentary succession in the eastern Anti-Atlas in the early Serpukhovian (Klug & Pohle, 2018; Klug et al., 2006).

## Material and methods

**Fig. 1:**
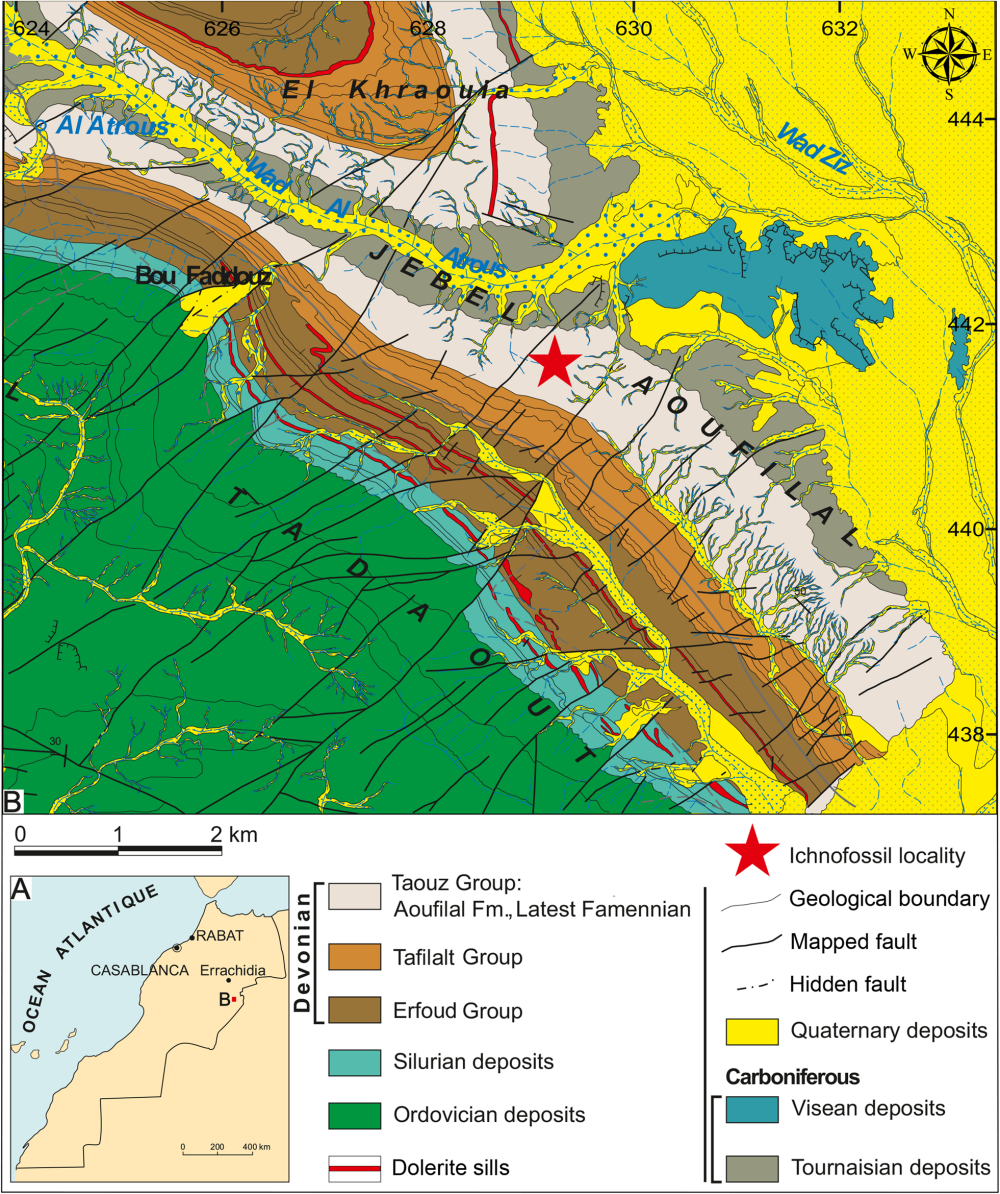
**A** Location map of the study area. **B** Geological map of Al Atrous region showing the trace-fossil locality (map extracted from Alvaro et al. (2014))

The single specimen described here was discovered and excavated by the miner and self-taught palaeontologist Mohamed Mezane near his home village El Khraouia. It is stored at the Paläontologische Institut und Museum der Universität Zürich with the number PIMUZ A/I 5060. The locality (N30.968449283419947, W-4.037403017377474) lies 1 km south of El Khraouia (also transcribed as Lahkraouia), about 15 km south of Merzouga and 7.8 km north northwest of Taouz in the southern Tafilalt (Fig. 1).

The specimen was photographed under white light from different directions to maximize the visibility of the swimming trace. Additionally, the specimen was 3D-scanned with an Artec Eva. The obtained data were processed with the software Artec 3D Studio 11.

## Geological setting

### Geomorphology

The ichnofossil slab was collected from a succession of finely clastic sediments, which is rich in trace fossils in this area. These sediments are fine-grained micaceous sandstones, which sometimes show some fine cross-bedding. The clay content varies, which is reflected in lateral and vertical changes in lamination and thickness of the single layers. They are under- and overlain by even finer clastics (clays and siltstones) and thus, they form elevations of strongly varying height and steepness. At the abandoned village Jebel Al Atrous, these strata form a rugged mountain, the Jebel Al Atrous (Tamazight language for ‘goat mountain’), while northeast of the Jebel Amessoui and south of El Khraouia, they merely form low hills. The geomorphological appearance as mountains in some places and very low hills in others is probably controlled rather by cementation and tectonics [dip, faults; cf. Baidder et al. (2016)] than by sedimentary thickness, since the thickness appears to change only slightly laterally.

### Stratigraphy

The Al Atrous area is dominated by the Ordovician claystone, sandstone and quartzite deposits of the Bani Group in the south-western part and the Devonian strata of the Erfoud, Tafilalt and Taouz Groups, which crop out in the centre of the Amessoui Syncline (Klug & Pohle, 2018). There, the Devonian strata overlie conformably the Silurian clayey to carbonatic deposits of the lower Erfoud Group in northwest and southeast direction. The Tournaisian and Visean (Oued Znaïgui and Merdani Formations) covers the Devonian strata unconformably (Alvaro et al., 2014; Benharref et al., 2014). The Hangenberg Black Shale and Sandstone equivalents belong to the Taouz Group.

**Fig. 2:**
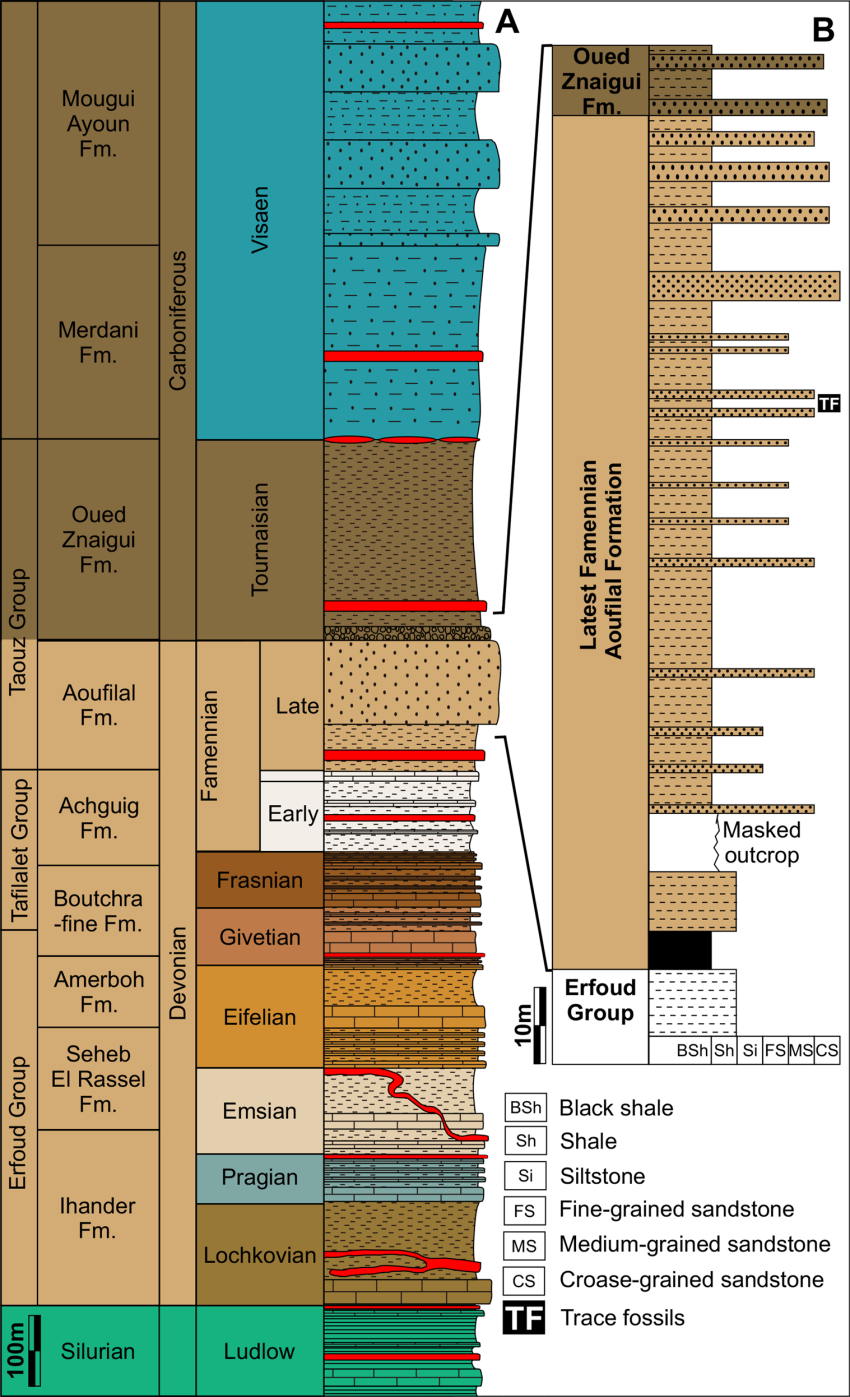
**A** Late Paleozoic stratigraphy subdivision of the South Tafilalt region, adapted from Álvaro et al. (2014) and Najih (2019); see more information on the log there. **B** Lithological section from the Al Atrous region with the stratigraphic position of ichnofossil-bearing strata

The Devonian deposits consist mainly of three formal stratigraphic units, which are from the base to the top: Lochkovian to Givetian Erfoud Group, Givetian to Middle Famennian Tafilalet Group, and the Late Famennian to Tournaisian Taouz Group (Fig. 2A). The latter is composed of the Late Famennian Aoufilal Formation and the Tournaisian Oued Znaigui Formation (Fig. 2A).

The stratigraphic position of the ichnofossil described here is somewhat obscured by the absence of index fossils, such as ammonoids or conodonts, nearby. Kaiser et al. (2013: p. 83) reported findings of *Acutimitoceras* (*Stockumites*) directly above the *Gonioclymenia* Limestone (Upper Member of the Tafilalt Group) at Al Atrous, which is about 5.5 km to the Northwest. Between El Khraouia and Al Atrous, the Devonian and Early Carboniferous strata are quite well exposed and can be traced easily on, e.g., satellite images. According to Kaiser et al. (2013), about 210 m of thin-bedded clastics overlie the *Gonioclymenia* Limstone unit, which is then overlain by the Hangenberg Sandstone lithostratigraphic equivalent, or at least its approximate correlate. At Al Atrous and El Khraouia, some tens to nearly 100 m higher in the sequence above the *Undichna*-bearing layer, ammonoid associations were documented by various authors (Becker et al., 2006; Kaiser et al., 2013; Korn et al., 2002, 2003). These are, however, already of Middle Tournaisian age. Only near the mine Mfis (about 6 km to the Northeast), an Early Tournaisian assemblage was documented (Bockwinkel & Ebbighausen, 2006). When correlating the dated sections of Kaiser et al. (2013) with the outcrop at El Khraouia, the *Undichna*-bearing layers likely correspond to the interval directly below the Devonian–Carboniferous boundary. This correlation was made based on the lithological correlation in the field, the geomorphological observation on satellite images and in the field, and the occurrences of index fossil-bearing layers, which, however, are quite far below and above the layer that yielded the ichnogenus *Undichna*. Accordingly, these clastic sediments at El Khraouia probably correlate with the Hangenberg limestone. In the Amessoui Syncline, the layers bearing *Undichna* and other ichnotaxa vary in facies and fossil content. At Al Atrous, brachiopods are quite abundant (Kaiser et al., 2013), while at El Khraouia, a well-preserved ichnofauna occurs in these layers (e.g., Lagnaoui et al., 2019).

### Sedimentology

**Fig. 3:**
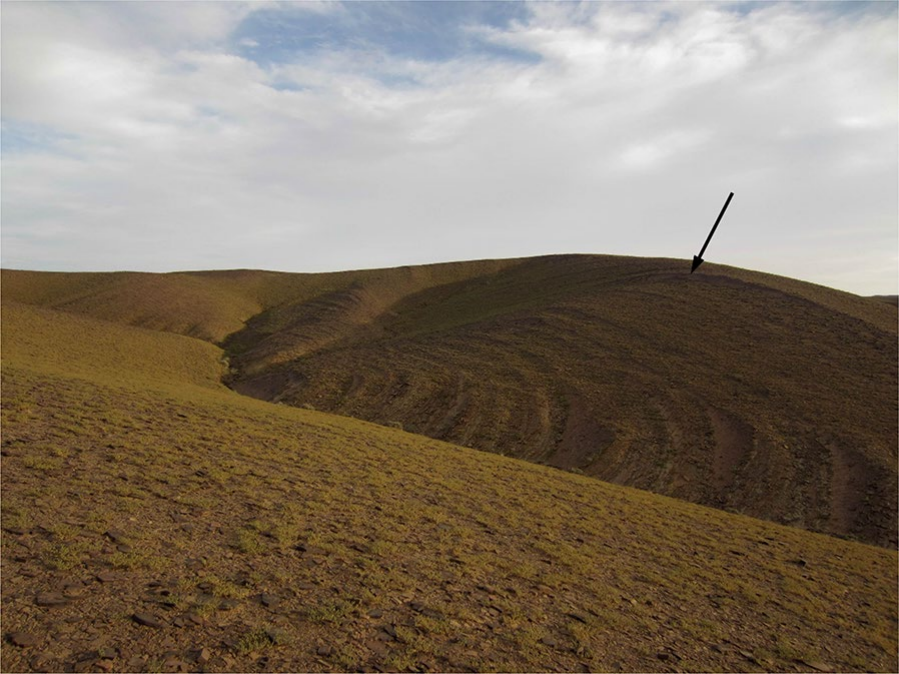
Photo of the outcrop with some vegetation after a reasonably rainy winter. Note the thin-bedded alternations of siltstone and fine-grained sandstones, which weather differentially relative to one another. Bed-thickness is likely due to variations in clay content, which, in turn, is probably controlled by variations in sea-level and clastic input. The *Undichna* described here is from the thicker bed marked by the arrow, which runs obliquely down the slopes on both sides of the arrow

The ichnotaxon *Undichna* described herein comes from the uppermost part of the Aoufilal Formation of the Taouz Group of latest Famennian age (see above). Between El Khraouia and Filon 12 (Jebel Aoufilal), the Aoufilal Formation rests locally directly on the Early Givetian deposits (Erfoud Group), (Figs. 1B, 2A). The locality is located on the Jebel Aoufilal-ridge, which is the type locality of this lithological unit. It comprises a thick series (about 260 m) of hardly fossiliferous black shales, mudstones, thin-bedded siltstones and fine- to medium-grained sandstones (field photo in Fig. 3) with occasional cross-bedding and ferruginous brown crusts, followed by a unit of sandstone rich in bioclasts. The top of the latter unit is characterised by one microconglomeratic bed intercalated with predominant sandstone layers rich in bioturbation and invertebrate trace fossils, which generally constitutes a level marked by detachment structures usually acting as slippery layers (Fig. 2B). The upper surfaces of the fine- to medium-grained sandstones preserve oscillation and current ripples, some sandstone layers show cross-bedding structures in the Tafilalt and Maïder regions.

## Results

**Fig. 4:**
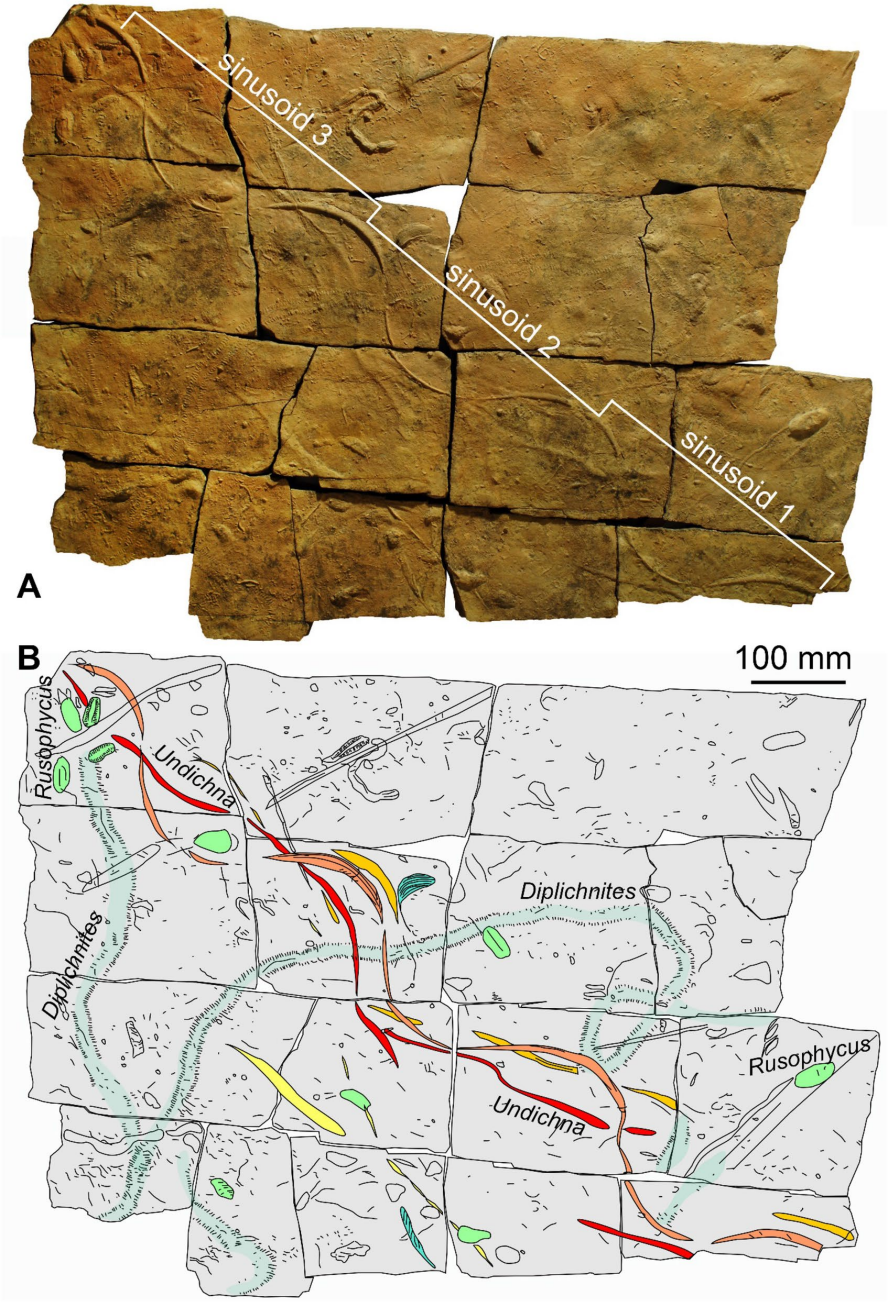
*Undichna britannica*, PIMUZ A/I 5060, equivalent of the Hangenberg sandstone, latest Devonian, El Khraouia, near Merzouga and Taouz. **A** Photo taken under shallowly angled white light with the main light source from the top right. **B** Drawing made from the photo in A with the main ichnofossils marked in colours (*Diplichnites*—light blue, *Rusophycus* light green). Those belonging to *U. britannica* are marked in dark orange (main trace), red, and light orange (two somewhat discontinuous secondary traces) while more doubtful parts running subparallel are marked in yellow. The dark orange part was likely produced by the caudal fin

**Fig. 5:**
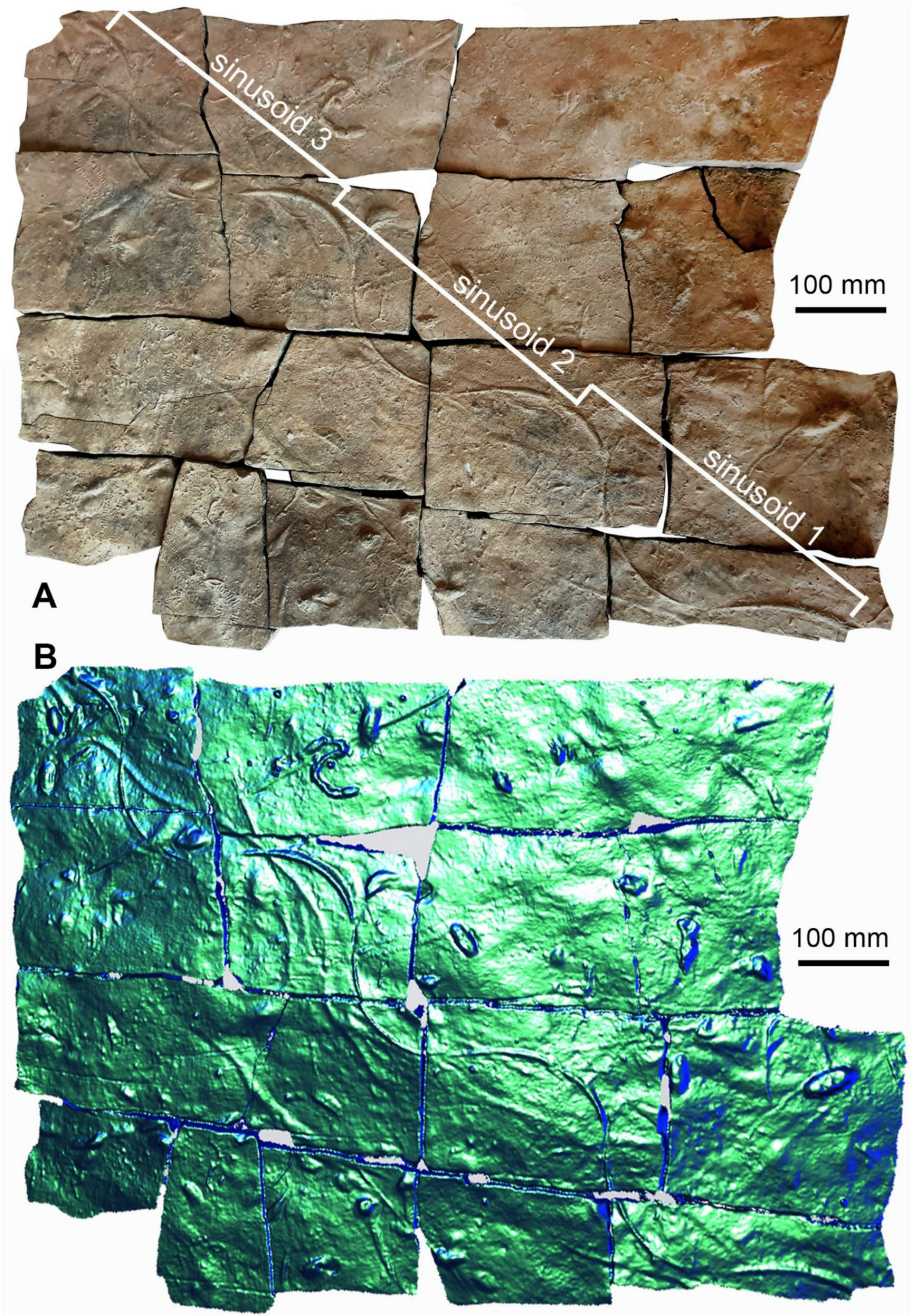
*Undichna britannica*, PIMUZ A/I 5060, equivalent of the Hangenberg sandstone, latest Devonian, El Khraouia, near Merzouga and Taouz. **A** Photo taken under shallow white light with the main light source on the bottom left. **B** 3D scan with Artec Eva. The tracks of the fins are well discernible, particularly the impression of the caudal fin is sharp, overlaying the others

The ichnofossil-bearing slab described here is about 0.98 m long and 0.69 m wide. It is broken into 16 subrectangular plates. The slab consists of a cross-bedded, fine-grained sandstone with a brownish colour. The ichnofossils are on the underside (lower bedding surface), while wave-generated ripple marks cover the upper bedding surface (not shown). The underside shows a wealth of ichnofossils including long *Diplichnites*, several *Rusophycus* and a few other ichnotaxa (Figs. 4, 5). Here, we focus on the *Undichna*, the entire ichnofauna is quite rich and will be described elsewhere.

The maximum length of the *Undichna* trace fossil is 1.05 m. There are several undulating convex hyporeliefs with a central main tail trace, which cuts through all other traces and carved the deepest into the sediment (marked in dark orange in Fig. 4B), which is evidenced by the latter being cut by the former. These traces are former grooves, now appearing as ridges in the hyporelief, which is up to 11 mm wide and maximally 3 mm deep (the infill is up to 3 mm high). The wavelength of the main furrow varies slightly and the three main sinusoids measure 323 mm (sinusoid 1), 322 mm (sinusoid 2), and 327 (sinusoid 3) mm in length (see Figs. 4A and 5A). The amplitude of the main furrow measure 80 mm (sinusoid 1), 87 mm (sinusoid 2), and 97 (sinusoid 3) mm. The main groove displays some very faint chevron patterning, suggesting a swimming direction from the top left to the bottom right in Figs. 4 and 5.

**Fig. 6:**
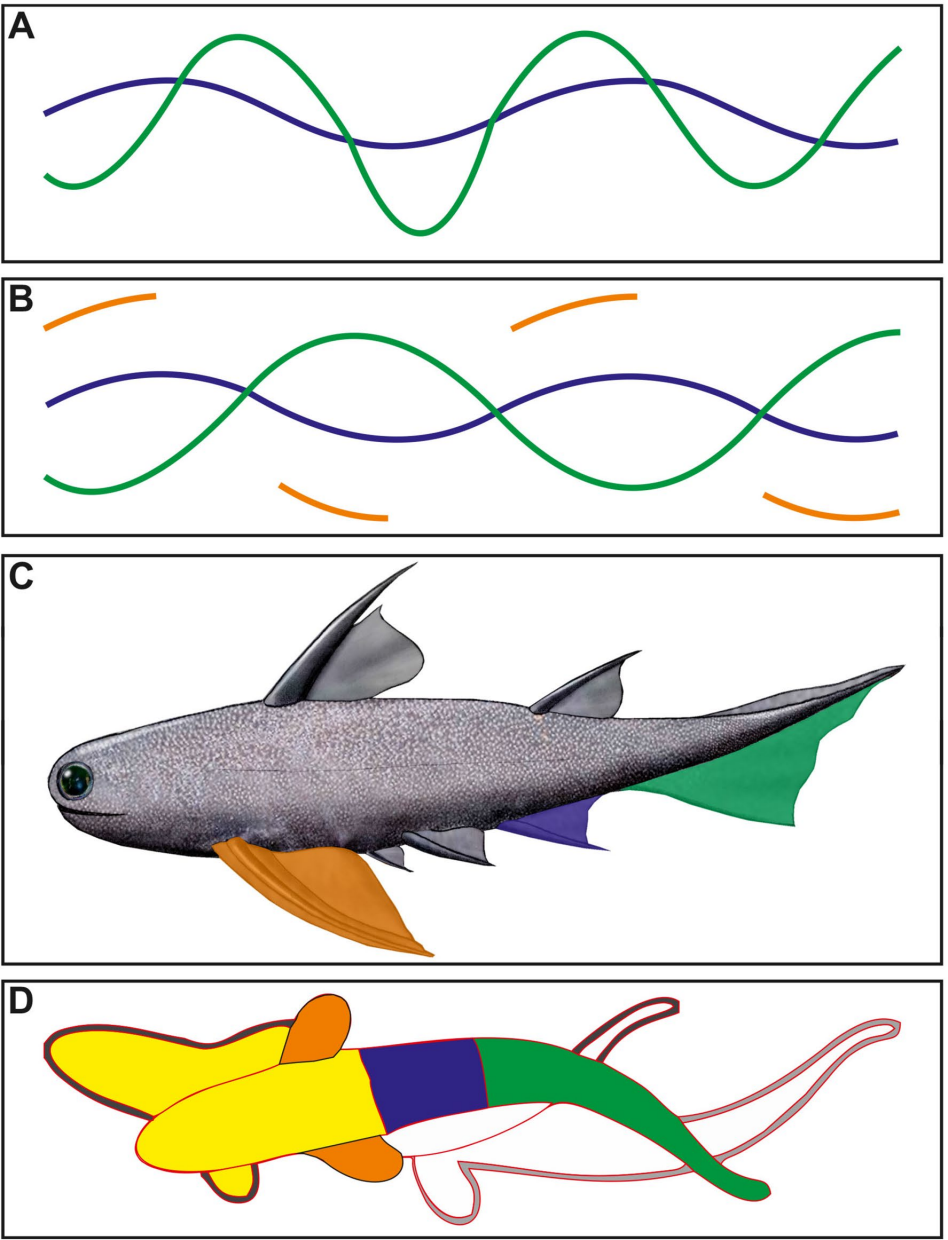
**A**, **B** Interpretative drawing of the two morphotypes of the ichnospecies *Undichna britannica.* **C** Schematic sketch of the anatomical features of a potential tracemaker (here an acanthodian as example). **D** Mode of movement based on the anatomical-controlled features recorded within the described *Undichna britannica* from Morocco

The secondary grooves undulate much less regularly (marked in red and light orange in Fig. 3). The median groove (marked in red in Fig. 4B and in blue in Fig. 6) shows a relatively narrow undulation in comparison with the marginal discontinuous secondary groove (marked in orange and yellow in Figs. 4B and 6). These two grooves can be traced across much of the plate and intersect with the main groove three times each. Their preservation is quite discontinuous, making it doubtful, in some cases, how they were previously and possibly linked to each other.

## Discussion

### Taxonomy

The main ichnotaxonomic features shown in the *Undichna* specimen of the Al Atrous area are the two out-of-phase, intertwined waves of different amplitude associated with a moderately narrow, sinuous, median furrow. These distinctive features match the diagnosis of *Undichna britannica* [trails of a paired fin alternate in the rhythm of undulation with that of the unpaired caudal fin; Minter and Braddy (2006)]. Other ichnospecies of *Undichna* differ in having several continuous grooves, paired main traces or else [see Minter and Braddy (2006) for an overview]. Our specimen best corresponds to the Figs. 2P, Q, U and V in Minter and Braddy (2006).

### Tracemaker and ethology

In the absence of distinct imprints of appendages, it appears unlikely that the trace fossil was produced by a tetrapod. Since placoderms became extinct at the Hangenberg Event, we can rule them out. Unfortunately, trace fossils of the paired fins are insufficiently distinct to determine whether a chondrichthyan or an osteichthyan produced the trace fossil. There is some weak indication suggesting chondrichthyan, because from the preceding Hangenberg Black Shale of the southern Maïder region, chondrichthyan remains are known (Klug et al., 2016). Actinopterygians and chondrichthyans are rarely reported from the Late Devonian of Morocco, only fragmentary remains have been published (Derycke, 1992, 2017; Derycke et al., 2008, 2014; Frey et al., 2018, 2019, 2020; Ginter et al., 2002; Klug et al., 2016; Lehman, 1976; Termier, 1936). Moreover, relatively similar swimming trace fossils assigned to *Undichna britannica* were described from the the Late Carboniferous of Spain, which was ascribed to Chondrichthyes (Soler-Gijon & Moratalla, 2001).

Remarkably, only the main groove shows a harmonious undulation, while the subordinate grooves produced by more anteriorly positioned fins sometimes cross the trace of the caudal fin and undulate much more irregularly. *U. britannica* is represented by two morphotypes. The first morphotype has a main trail trace with high-amplitude undulation and a relatively narrow undulation of the median trail trace and the second one has a main trail trace with a relatively medium amplitude undulation and a rather narrow median trail trace (Trewin, 2000). *Undichna britannica* has been commonly interpreted by a subcarangiform swimming behaviour (Cardonatto and Melchor, 2014).

The subcarangiform swimmers use only the rear two-thirds of their bodies to generate thrust (Cardonatto and Melchor, 2014), keeping the anterior third comparatively still (Fig. 6). The narrow undulation is probably produced by relatively narrow undulate movement of the anal fin (blue colour in Fig. 6). However, the main trail trace with a rather constant undulation is produced by the movement of the caudal fin, where the majority of the work of displacing water is done (green colour in Fig. 6). Moreover, the pelvic fins were probably in discontinuous contact with the sediment surface; this would explain the discontinuous nature of the lateral trail traces (orange blue colour in Fig. 6). This form of swimming increases speed by concentrating the lateral movements towards the posterior end of the body. This behaviour might be developed for escaping predators or even chasing down prey. Therefore, the green part of the tracemaker’s body in Fig. 6D moves completely in an undulation movement, the blue part of the tracemaker’s body (Fig. 6D) produces a slightly undulate movement, while the yellow part of the tracemaker’s body is mostly a sub-static part.

Bainbridge (1958), Videler (1993) as well as Wisshak et al. (2004) attempted to estimate body size of the tracemaker based on the dimensions of the grooves and its undulations. They suggested that one wavelength of the trace of the caudal fin should be around 67% of the body length, or five times the amplitude of the trace of the caudal fin. Accordingly, the body of the tracemaker would have been about 485 mm long when using the wavelength or between 400 and 485 mm when using the variation of the amplitude of the main trace. Accordingly, the fish was about half a metre long, which appears to be a reasonable estimate for a non-placoderm fish of the latest Famennian.

### Palaeoenvironment

The ichnospecies *Undichna britannica* is more commonly recorded in lacustrine and estuarine settings. Nevertheless, it has been recorded in fluvial and other shallow marine environments (Cardonatto and Melchor, 2014; Minter & Braddy, 2006).

The fine clastic sediment and the wave-induced ripple marks point at a shallow water environment in the area. The reasonable abundance of starfish resting traces prove that the environment was marine (e.g., Klug & Pohle, 2018: fig. 17). This is also supported by the presence of cephalopods in layers tens of metres above and below the ichnofossil-bearing layer (Kaiser et al., 2013).

The presence of resting traces of starfish and various arthropods as well as walking traces of arthropods [Klug and Pohle (2018: fig. 17); a more detailed account will be published in the next years] allow to assign this layer to the *Cuziana* Ichnofacies (e.g., Seilacher, 1964, 2007). According to MacEachern et al. (2012: tab. 1), this suggests “low energy”, “food deposited on or buried in the sediment”, “subtidal settings lying far below fair-weather wave base but above storm wave base”. Moreover, the transition from thin-bedded, olive-green siltstones and shales to fine-grained to medium-grained sandstones and conglomerate indicate a change from deeper water shallowing upward into a low-energy depositional environment (e.g., Kaiser et al., 2018).

### Vertebrate evolution

Following the Hangenberg Event, the latest Devonian and Early Carboniferous represented a bottle neck in vertebrate evolution (Sallan & Coates, 2010; Sallan & Galimberti, 2015). After the Hangenberg Event, it appears that eco-space was vacated through the extinction of placoderms and, to a lesser degree, by the diversity decrease among sarcopterygians (Sallan & Coates, 2010). Our conclusion that the tracemaker is likely either a small chondrichthyan or an actinopterygian fits quite well with the accelerated radiation of both groups after the Hangenberg mass extinction.

## Conclusions

We describe the first record of *Undichna* from the Devonian of Gondwana. Its shape is typical of the ichnospecies *U. britannica*. We could not identify the tracemaker with certainty, but the presence of chondrichthyan remains in the only slightly older Hangenberg Black Shale in the eastern Anti-Atlas suggests that it might have been a chondrichthyan that produced this trace fossil. Based on the dimensions of the trace fossil, we assume that the animal was about half a metre long. The occurrence of a chondrichthyan or an osteichthyan directly following this mass extinction corroborates the hypothesis that at least parts of these two clades profited from the mass extinction and underwent a radiation in its wake. We plan further exploration in the region for body- and trace-fossils of fishes to refine the trace–tracemaker correlation and to contribute to the understanding of evolution of Late Devonian palaeo-ecosystems before and after the Hangenberg crisis.

## Data Availability

The single specimen is stored in the collections of the Palaeontological Institute and Museum of the University of Zurich (PIMUZ A/I 5060). The surface scan is available at www.figshare.com with the https://doi.org/10.6084/m9.figshare.16539567.
